# Synovial fluid-derived extracellular vesicles modulate joint cell function in osteoarthritis

**DOI:** 10.3389/fragi.2026.1894052

**Published:** 2026-09-30

**Authors:** Alvaro Compañ, Vittoria Carrabs, María Luisa Ferrándiz, Antonio Silvestre, Ignacio Muñoz, María Carmen Carceller, María Isabel Guillén

**Affiliations:** 1 Interuniversity Research Institute for Molecular Recognition and Technological Development (IDM), Universitat Politècnica de València, Universitat de València, Valencia, Spain; 2 Department of Pharmacy, University CEU Cardenal Herrera, Valencia, Spain; 3 Department of Pharmacology, University of Valencia, Valencia, Spain; 4 Service of Orthopedic Surgery and Traumatology, University Clinical Hospital, Valencia, Spain; 5 Hospital Ribera IMSKE, Valencia, Spain

**Keywords:** aging, chondrocytes, extracellular vesicles, hyaluronic acid, matrix metalloproteinases, osteoarthritis, synovial fluid, synoviocytes

## Abstract

**Introduction:**

Osteoarthritis (OA) is an age-related joint disease characterized by chronic inflammation and extracellular matrix degradation. Extracellular vesicles (EVs) in synovial fluid (SF) may modulate intercellular communication and influence disease progression.

**Material and methods:**

SF, cartilage, and synovial tissue were collected from knee OA patients (n = 68). SF-derived EVs (SF-EVs) were isolated by size exclusion chromatography and characterized by transmission electron microscopy (TEM), tunable resistive pulse sensing (qNano), and ELISA analysis of EV-associated markers (CD9, CD81, TSG101, and PDCD6IP) and the negative marker calnexin. *In vitro* experiments were performed using primary synoviocytes and chondrocytes stimulated with interleukin (IL)-1β in the presence or absence of SF-EVs. Inflammatory and catabolic markers were measured.

**Results:**

SF-EVs (∼261 nm; ∼7 × 10^8^ particles/mL) expressed canonical EV markers. In synoviocytes, SF-EVs reduced IL-1β-induced proliferation and decreased IL-6 and MMP-3 while increasing hyaluronic acid (HA). In chondrocytes, SF-EVs reduced IL-6 and MMP-13 and enhanced HA and IL-1Ra production, with sustained effects over time.

**Conclusion:**

These findings suggest that SF-EVs can modulate inflammatory and matrix-related responses in joint-resident cells under IL-1β-induced inflammatory conditions. Their distinct effects on synoviocytes and chondrocytes highlight the potential role of SF-EVs in regulating cell-specific responses within the osteoarthritic joint and warrant further investigation into their contribution to OA pathophysiology.

## 1 Introduction

Osteoarthritis (OA) is a chronic degenerative joint disease that significantly reduces patients’ quality of life (Kim, 2022). Its prevalence has risen markedly over recent decades, largely driven by population ageing, as advanced age represents one of the most significant risk factors for both the development and progression of OA (Courties et al., 2024). Aging promotes structural and functional changes in joint tissues, including extracellular matrix (ECM) degradation, chondrocyte senescence, impaired subchondral bone remodeling, and chronic low-grade inflammation, all of which contribute to the onset and exacerbation of knee OA (Sanchez-Lopez et al., 2022; Jang et al., 2021; Motta et al., 2022). The mechanisms underlying cartilage degradation have been extensively investigated. A prevailing hypothesis suggests that ECM degradation products contribute to disease progression by promoting receptor-mediated activation of synoviocytes and chondrocytes, leading to chronic inflammation (Homandberg et al., 1992; Yasuda and Poole, 2002; Yasuda et al., 2003; Werb et al., 1989). Under physiological conditions, chondrocytes regulate cartilage structure through the synthesis of collagen type II and other ECM components, while synoviocytes produce synovial fluid (SF), a specialized lubricant essential for joint function. In OA, this balance is disrupted: inflammatory mediators and mechanical stress drive chondrocytes toward a catabolic phenotype characterized by the release of cytokines, including interleukin-1β (IL-1β) and tumor necrosis factor-α (TNF-α), as well as matrix-degrading enzymes such as matrix metalloproteinases (MMPs) and disintegrin and metalloproteinase with thrombospondin motifs 5 Type 1 (ADAMTS-5), which are primarily responsible for cartilage degradation (Murphy et al., 2018; Glasson et al., 2005; Kellgren and Lawrence, 1957). These alterations are reflected in changes in SF composition. SF is enriched in synoviocyte-secreted components such as hyaluronic acid (HA), lubricin, phospholipids, and glycosaminoglycans, which are essential for lubrication, shock absorption, and joint homeostasis (Fallacara et al., 2018; More et al., 2020; Dahl et al., 1985). During OA, osteoarthritic synovial fluid (OSF) undergoes marked changes, including reduced HA molecular weight, accumulation of catabolic enzymes, and increased levels of inflammatory mediators (Fallacara et al., 2018; More et al., 2020; Dahl et al., 1985; Volpi et al., 2009; Fraser et al., 1997).

Recent studies have further highlighted the role of extracellular vesicles (EVs) in joint degeneration. EVs released from primary chondrocytes of OA patients, particularly following IL-1β stimulation, exert detrimental effects on healthy chondrocytes (Guo et al., 2021). Similarly, long non-coding RNA (lncRNA) PCGEM1 carried by EVs derived from OA synoviocytes, promotes IL-1β expression, leading to chondrocyte apoptosis and matrix degradation. Chondrocytes can also internalize EVs derived from OA subchondral osteoblasts enriched in microRNA (miR)-210-5p, which reduces oxygen consumption and induces catabolic gene expression (Wu et al., 2021). In addition, EVs from patients with knee OA promote joint degeneration by recruiting inflammatory cells, inhibiting cartilage repair, and increasing inflammatory marker expression (Gao et al., 2020). EVs enriched in miR-193b-3p, targeting HDAC3 and promoting histone H3 acetylation, have also been shown to modulate chondrocyte catabolism (Meng et al., 2018). Collectively, these findings indicate that EVs from different cellular sources can have different roles and context-dependent roles in OA progression, contributing to cartilage degradation, inflammation, and the disruption of joint homeostasis. Within this context, synovial fluid-derived EVs (SF-EVs) represent key regulators of intercellular communication in the joint. They transport a wide range of bioactive cargo, including non-coding RNAs, metabolites, cytokines, growth factors, and proteases, influencing molecular crosstalk between joint tissues and immune cells (Gibbings et al., 2009; Robbins et al., 2016). Importantly, the production, cargo composition, and functional impact of EVs are modulated by aging, cellular senescence, and inflammation, which progressively reshape the joint environment (Wu et al., 2020). There is increasing evidence that SF-EVs have a direct impact on both chondrocytes and synoviocytes. Notably, EVs can exert both pro-inflammatory and anti-inflammatory effects depending on their origin and molecular cargo. SF-EVs isolated from osteoarthritic joints promote inflammatory responses in chondrocytes and macrophages (Zhang et al., 2023), while EVs released by activated monocytes induce the expression of inflammatory markers and markers of ECM degradation in joint cells (Carrabs et al., 2024). Conversely, mesenchymal stem cell-derived EVs exhibit anti-inflammatory and chondroprotective properties by suppressing cytokine release and inhibiting catabolic pathways (Alcaraz et al., 2019). This dual behaviour highlights the incompletely understood role of EVs in inflammatory regulation and OA pathogenesis, particularly in the aging joint. Considering the dynamic nature of SF, aging-related alterations in joint homeostasis, and the context-dependent effects of EV-mediated signaling, this study aimed to investigate the role of SF-EVs from patients with OA in the modulation of joint cell function, especially in synoviocytes and chondrocytes.

## 2 Materials and methods

### 2.1 Participant selection and human sample collection

Synovial tissue, cartilage, and synovial fluid samples were obtained from 68 patients diagnosed with knee osteoarthritis undergoing joint replacement surgery. The mean age of the participants was 67 years (range 50–83 years) with a female predominance (54%). All patients underwent total knee replacement for advanced knee osteoarthritis. Seventeen samples were excluded because they were obtained from patients diagnosed with rheumatoid arthritis or traumatic joint injury, or due to technical reasons such as insufficient sample volume, haemolysis or contamination of synovial fluid, or failed cell culture. Consequently, the final study included 25 synovial fluid samples, 14 synovial tissue samples, and 12 articular cartilage samples, which were used in the different experimental procedures according to sample availability. The number of independent biological samples included in each experiment is indicated in the corresponding figure legends. Synovial fluid samples were macroscopically evaluated for appearance and viscosity. In addition, biochemical analyses were performed to determine C-reactive protein (CRP), interleukin-1β (IL-1β), interleukin-6 (IL-6), and hyaluronic acid (HA) levels for sample characterization. Samples were collected from the Hospital Clínico Universitario of Valencia and Instituto Musculoesquelético Europeo of Valencia. This study was conducted following the Declaration of Helsinki and approved by the Human Research Ethics Committee of Hospital Clínico Universitario de Valencia, Spain (No. 2023/101; Protocol version 3, 12 June 2023; HIP/CI: version 2, 12 June 2023) and the Institutional Ethical Committee of the University of Valencia (No. H1512058528966). Written informed consent was obtained from all participants prior to sample collection.

### 2.2 Isolation and culture of human osteoarthritic synoviocytes

Human synovial membrane samples were collected aseptically from knee joint specimens obtained during total joint replacement surgery in patients with osteoarthritis. Samples were immediately immersed in Dulbecco’s Modified Eagle’s Medium (DMEM, Sigma-Aldrich) supplemented with 1% penicillin-streptomycin (Gibco, Thermo Fisher Scientific, Waltham, MA, United States of America) and transported to the laboratory on ice. Within 24 h, visible adipose tissue was carefully removed using sterile forceps and scissors. The synovial tissue was finely minced (<2 mm fragments) and transferred to 50 mL conical centrifuge tubes containing 9 mL of DMEM with antibiotics and 1 mL of collagenase IA (Sigma-Aldrich). Enzymatic digestion was performed at 37 °C with orbital agitation (100 rpm) for 2–3 h to disaggregate the tissue. Following digestion, the sample was filtered sequentially through 100 μm and 70 μm nylon mesh filters (Corning) under sterile conditions, and the remaining cells were rinsed with DMEM supplemented with 10% fetal bovine serum (FBS, Biowest). The collected suspension was centrifuged at 272 *g* for 10 min at room temperature, the supernatant was discarded, and the pellet was resuspended in DMEM containing 10% FBS. Cells were counted using the Trypan Blue exclusion method in a Neubauer chamber and cultured at 37 °C in a humidified incubator with 5% CO_2_, with medium changes every other day until reaching confluence.

Synoviocytes at passage one were selected for experimental use, as viability and responsiveness were optimal at this stage. For cryopreservation, trypsinized cells were resuspended in FBS containing 10% dimethyl sulfoxide (DMSO) and stored at - 80 °C or in liquid nitrogen for long-term preservation.

#### 2.2.1 Experimental treatment

For stimulation assays, synoviocytes were seeded at 30,000 cells/mL in 96-well plates (100 μL/well; 3,000 cells/well), 100,000 cells/mL in 12-well plates (0.5 mL/well; 50,000 cells/well), and 200,000 cells/mL in 6-well plates (1 mL/well). Culture medium was replaced with fresh culture medium supplemented with 10% EV-depleted FBS and containing recombinant human IL-1β (10 ng/mL, PeproTech). This concentration was selected based on previous studies from our group demonstrating a robust and reproducible inflammatory response in primary human osteoarthritic synoviocytes (García-Arnandis et al., 2010; García-Arnandis et al., 2011). The selected SF-EV concentrations were chosen to evaluate a broad range of biologically relevant particle concentrations and are consistent with concentrations previously used in in vitro studies investigating the biological effects of EVs on joint cells (Carrabs et al., 2024; Guillén et al., 2021). Cells were treated in the presence or absence of SF-EVs at concentrations of 1.8 × 10^7^, 7.2 × 10^7^, or 7.2 × 10^8^ particles/mL, for 48 or 96 h. After incubation, supernatants were centrifuged at 370 *g* for 5 min at 4 °C and stored at - 80 °C for subsequent analysis.

### 2.3 Isolation and culture of human osteoarthritic chondrocytes

Cartilage samples were obtained from the same osteoarthritic patients during surgery, immediately washed in DMEM with 1% penicillin-streptomycin and macroscopically inspected to ensure absence of visible calcifications or contamination. Cartilage was separated from attached soft tissue using a sterile scalpel, minced, and enzymatically digested following a protocol like that used for synoviocytes (1 mL collagenase in 9 mL DMEM) under agitation at 37 °C for 2–3 h.

The resulting suspension was filtered through 70 μm mesh, and residual fragments were rinsed with complete DMEM medium. After centrifugation at 272 *g* for 10 min, the chondrocyte pellet was resuspended in DMEM supplemented with 10% FBS. Cell counts and viability assessment were performed as described above, and chondrocytes were cultured at 37 °C in a humidified incubator with 5% CO_2_, with medium changes every other day until reaching confluence.

Passage 0 chondrocytes were used for all experiments to preserve their native phenotype.

#### 2.3.1 Experimental treatment

For stimulation assays, chondrocytes were seeded at 30,000 cells/mL in 96-well plates (100 μL/well; 3,000 cells/well), 100,000 cells/mL in 12-well plates (0.5 mL/well; 50,000 cells/well), and 200,000 cells/mL in 6-well plates (1 mL/well). Culture medium was replaced with fresh culture medium supplemented with 10% EV-depleted FBS and containing recombinant human IL-1β (10 ng/mL). This concentration was selected based on our previous studies in primary human osteoarthritic chondrocytes, in which this concentration consistently induced a robust inflammatory response (Carrabs et al., 2024; Guillén et al., 2021; Platas et al., 2013). Chondrocytes were treated in the presence or absence of SF-EVs at the same final concentrations described for synoviocytes (1.8 × 10^7^, 7.2 × 10^7^, and 7.2 × 10^8^ particles/mL) for 48 or 96 h at 37 °C. After treatment, supernatants were collected, centrifuged at 300 *g* at 4 °C and stored at −80 °C until subsequent analysis. The overall experimental workflow of the study is summarized in Scheme 1.

**SCHEME 1:**
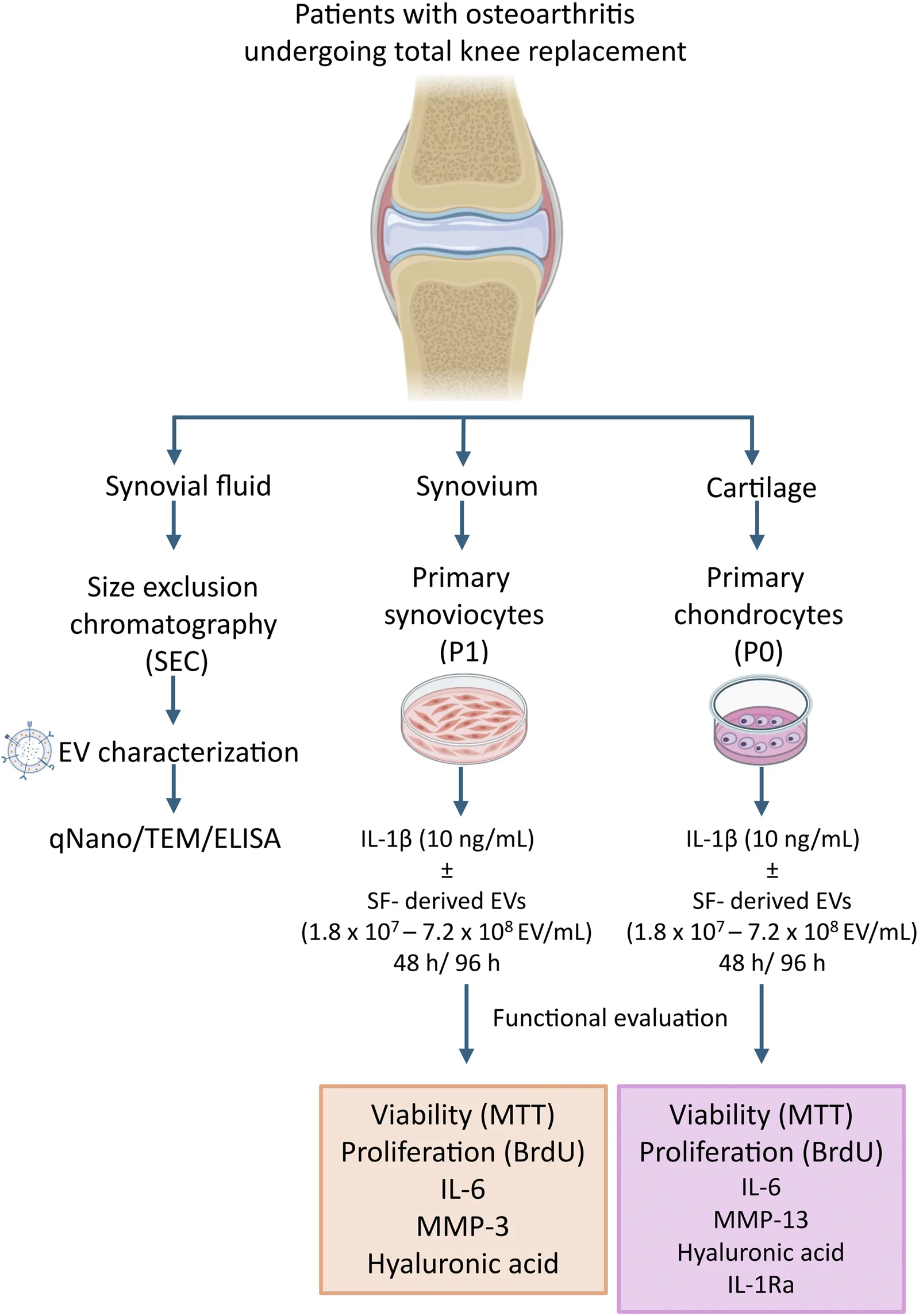
Experimental workflow of the study. Synovial fluid-derived extracellular vesicles (SF-EVs) were isolated from osteoarthritic synovial fluid by size exclusion chromatography (SEC), characterized by tunable resistive pulse sensing (TRPS; qNano), transmission electron microscopy (TEM), and ELISA, and subsequently evaluated in IL-1β-stimulated primary osteoarthritic synoviocytes and chondrocytes. Cell proliferation, inflammatory mediators, catabolic markers, hyaluronic acid, and IL-1Ra production were assessed. Created in BioRender. Carceller Zazo, M. D. C. (2026) https://BioRender.com/cutl85t.

### 2.4 Isolation and purification of osteoarthritic synovial fluid extracellular vesicles (EVs)

Osteoarthritic synovial fluid (OSF) was aspirated intraoperatively using sterile syringes and transferred to 1.5 mL tubes. Samples were centrifuged at 10,000 × g for 10 min at 4 °C to remove cells and large debris. Supernatants were aliquoted in cryovials with unique patient/sample codes and stored at −80 °C until further processing.

Thawed synovial fluid was filtered through 0.22 μm polyvinylidene fluoride (PVDF) filters and subjected to sequential size exclusion chromatography (SEC) using Exo-spin™ midi columns (Cell Guidance Systems, EX04) or Izon qEV2 columns (IC2-35, Izon Science) according to the manufacturers’ instructions. Both SEC-based purification platforms are designed to enrich EVs while reducing the co-isolation of soluble proteins and other low-molecular-weight contaminants.

Columns were pre-equilibrated with filtered phosphate-buffered saline (PBS), and the sample was loaded according to the manufacturer’s instructions. Fractions 7–16, corresponding to the EV-enriched elution window recommended by the manufacturers, were pooled to maximize extracellular vesicle recovery while minimizing the co-elution of soluble proteins and other low-molecular-weight contaminants. The pooled fractions were aliquoted and stored at −80 °C until use. All procedures were performed avoiding repeated freeze–thaw cycles to preserve vesicle integrity. Subsequent EV characterization is described in Section 2.5.

Because the operating theatre protocol did not allow the simultaneous collection of synovial fluid together with synovial tissue or cartilage from the same patient, EVs and recipient cells were obtained from different donors (allogeneic experimental design). Due to the limited amount of EVs recovered from individual synovial fluid samples, EV preparations were pooled before functional assays. This strategy provided sufficient material for EV characterization and ensured the use of the same EV preparation throughout all cellular assays, thereby improving experimental reproducibility. The number of independent biological samples (n) used in each experiment is indicated in the corresponding figure legends.

### 2.5 Characterization of extracellular vesicles

#### 2.5.1 Tunable resistive pulse sensing (TRPS)

EV concentration, size distribution, and surface charge (zeta potential) were measured using the qNano instrument (Izon Science Ltd.), following supplier protocols. The instrument was equipped with an NP150 nanopore (Izon, NP150, designed for detection of vesicles between 70–420 nm). Calibration was performed with polystyrene calibration beads of defined size and concentration (CPC200, Izon). Samples were suspended in PBS with 0.05% Tween 20 to reduce aggregation. Data collection focused on getting at least 500 particles per sample to ensure statistical validity, and analysis included histograms of particle diameters and calculation of mean/mode size, as well as zeta potential estimation.

#### 2.5.2 Transmission electron microscopy (TEM)

EVs morphology and ultrastructure were evaluated by transmission electron microscopy (TEM) at the Central Service for Experimental Research, University of Valencia. Preparation involved concentration with Izon’s qEV concentration kit, chemical fixation, and cryosectioning. Immunogold labelling was used with anti-CD63 primary antibodies with gold nanoparticle-conjugated secondary antibodies to confirm exosomal marker presence. Digital captures were obtained at 25,000× and 40,000× magnification using a Hitachi HT7800 TEM (100 kV). Images were analysed for vesicle size, shape, membrane characteristics, and labelling specificity.

#### 2.5.3 Phenotypic analysis by ELISA

The following EV-enriched surface and cytosolic protein markers were analyzed: the tetraspanins (CD9 and CD81) (Cell Guidance Systems, Cambridge, United Kingdom; Cat #EX101 and Cat #EX103); the endosomal sorting complexes required for transport (ESCRT)-associated protein tumor susceptibility gene 101 (TSG101) (Aviva Systems Biology, San Diego, CA, United States of America; Cat#OKCA02151); the programmed cell death six-interacting protein (PDCD6IP) (Wuhan Fine Biotech Co., Wuhan, China; Cat #EH10966). Calnexin (CNX) was included as a negative marker to assess the presence of intracellular membrane-derived contaminants in the EV preparations (ABclonal, Wuhan, China; Cat #RK01142). All proteins were assessed by enzyme-linked immunosorbent assay (ELISA) using commercially available kits according to the manufacturers’ instructions. Samples were analyzed using a Victor 3™ V1420 Multilabel Counter (PerkinElmer España, Madrid, Spain).

### 2.6 Functional assays

#### 2.6.1 Cell viability (MTT assay)

Synoviocytes and chondrocytes subjected to experimental treatments were assessed for viability using the 3-(4,5-dimethylthiazol-2-yl)-2,5-diphenyltetrazolium bromide (MTT) assay (Sigma-Aldrich). At defined time points (24, 48, 72, 96 h), culture media was replaced with freshly prepared MTT solution (0.5 mg/mL in DMEM), incubated at 37 °C for 1–3 h. Formazan crystal formation was visualized by microscopy, then dissolved in dimethyl sulfoxide (DMSO), and absorbance recorded at 531 nm using a Victor3™ V1420 Multilabel Counter. Cell viability was expressed as a percentage of the control.

#### 2.6.2 Cell proliferation (BrdU incorporation)

Cell proliferation was determined by using the 5-bromo-2′-deoxyuridine (BrdU) incorporation ELISA (Abcam, Cambridge, United Kingdom; Cat #ab126556). Following experimental incubation, BrdU was added to cultures, which were then fixed, permeabilized, and processed for antibody detection according to the manufacturer’s instructions. Absorbance was measured at 450 nm and used as an index of relative DNA synthesis.

#### 2.6.3 Determination of inflammatory mediators in synovial fluid, and synoviocytes and chondrocytes supernatants

Concentrations of IL-1β (R&D Systems, Minneapolis, MN, United States of America; Cat #DY201), IL-6 and C-reactive protein (CRP) (ABclonal, Wuhan, China; Cat #RK00004 and RK00078), hyaluronic acid (HA) (Wuhan Fine Biotech Co., Wuhan, China; Cat #EU2556), matrix metalloproteinase-3 (MMP-3), and matrix metalloproteinase-13 (MMP-13) (Thermo Fisher Scientific, Waltham, MA, United States of America; Cat #BMS20143 and Cat #EHMMP13) were quantified by ELISA using commercially available kits, according to the manufacturers’ instructions.

IL-1β, IL-6, CRP, and HA were measured in synovial fluid samples, whereas IL-6 and HA were additionally quantified in culture supernatants from synoviocytes and chondrocytes in the presence or absence of SF-derived EVs. MMP-3 was measured in synoviocyte supernatants and MMP-13 in chondrocyte supernatants.

Absorbance was measured using a Victor3™ V1420 Multilabel Counter.

### 2.7 Statistical analysis

All data are expressed as median (IQR), unless otherwise indicated. The number of biological replicates in each experiment is indicated as “n” in the corresponding figure legends or text. Statistical differences among multiple experimental groups were assessed using the Kruskal-Wallis test followed by Dunn’s multiple comparisons *post hoc* test. Statistical analysis and data visualization were conducted using GraphPad Prism version 8.0 (GraphPad Software, San Diego, CA, United States of America). A p-value <0.05 was considered statistically significant.

## 3 Results

A total of 68 patients were initially recruited. Following patient selection and sample quality assessment, 25 synovial fluid samples, 14 synovial tissue samples, and 12 articular cartilage samples were available for the different analyses according to sample availability.

### 3.1 Characterization of osteoarthritic synovial fluid

Osteoarthritic synovial fluid (OSF) samples were macroscopically evaluated for appearance, including transparency, viscosity, and colour. A total of 25 synovial fluid samples were EV isolation and characterization. The samples were transparent to slightly turbid, yellowish in colour, and exhibited medium-to-high viscosity. Baseline concentrations of C-reactive protein (CRP), interleukin (IL)-1β, IL-6, and hyaluronic acid (HA) were quantified by ELISA to characterize the inflammatory and biochemical profile of the samples prior to EV isolation. The results are summarized in Supplementary Table S1. Overall, the samples showed low CRP levels together with detectable IL-1β and IL-6 concentrations and high HA levels, consistent with the characteristics of osteoarthritic synovial fluid.

### 3.2 Characterization of extracellular vesicles from OSF

EVs were isolated and purified from the OSF samples as previously described in the Materials and Methods section. EV size, concentration, and electrical charge were analyzed using qNano (Figure 1). TRPS analysis of 25 independent EV preparations (n = 25) showed a mean particle size of 261.4 ± 26.7 nm, with a mode of 196.4 ± 21.9 nm. The complete particle size distribution shown in Figure 1 indicates a heterogeneous EV-enriched population with a broad size range. The mean particle concentration was 7 × 10^8^ ± 1.7 × 10^8^ particles/mL. In addition, the EV-enriched preparations exhibited a net negative surface charge of approximately −20 mV.

**FIGURE 1:**
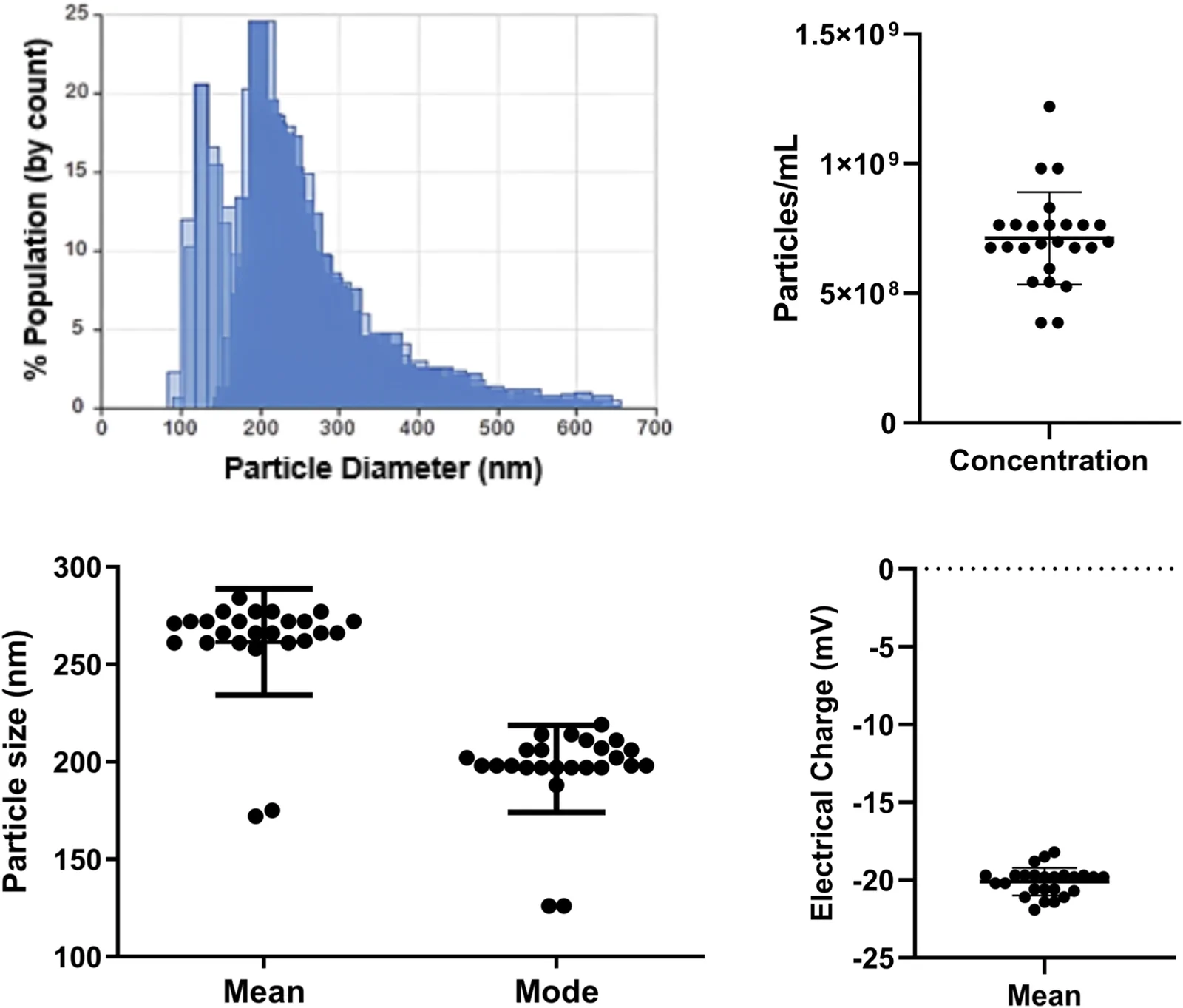
Characterization of EVs isolated from OSF. Representative particle analysis using qNano technology. Concentration of EVs (particles/mL) present in the OSF of patients with OA. Mean size and mode of EVs in the OSF of patients with OA. Mean electric charge of EVs in the analyzed OSF samples. n = 25.

To characterize the purified EV-enriched preparations, we evaluated the presence of EV-associated surface markers (CD9 and CD81), cytosolic markers (TSG101 and PDCD6IP) and the endoplasmic reticulum protein CNX as a negative marker by ELISA (Figure 2). The median (IQR) concentration in OSF found for CD9 and CD81 were of 3.86 (2.35–6.08) µg/mL and 3.22 (2.14–4.03) µg/mL, respectively. The median (IQR) for TSG101 and PDCD6IP were 133.80 (107.56–242.06) µg/mL and 180.61 (133.96–266.97) µg/mL, respectively. The CNX protein was undetectable (data not shown).

**FIGURE 2:**
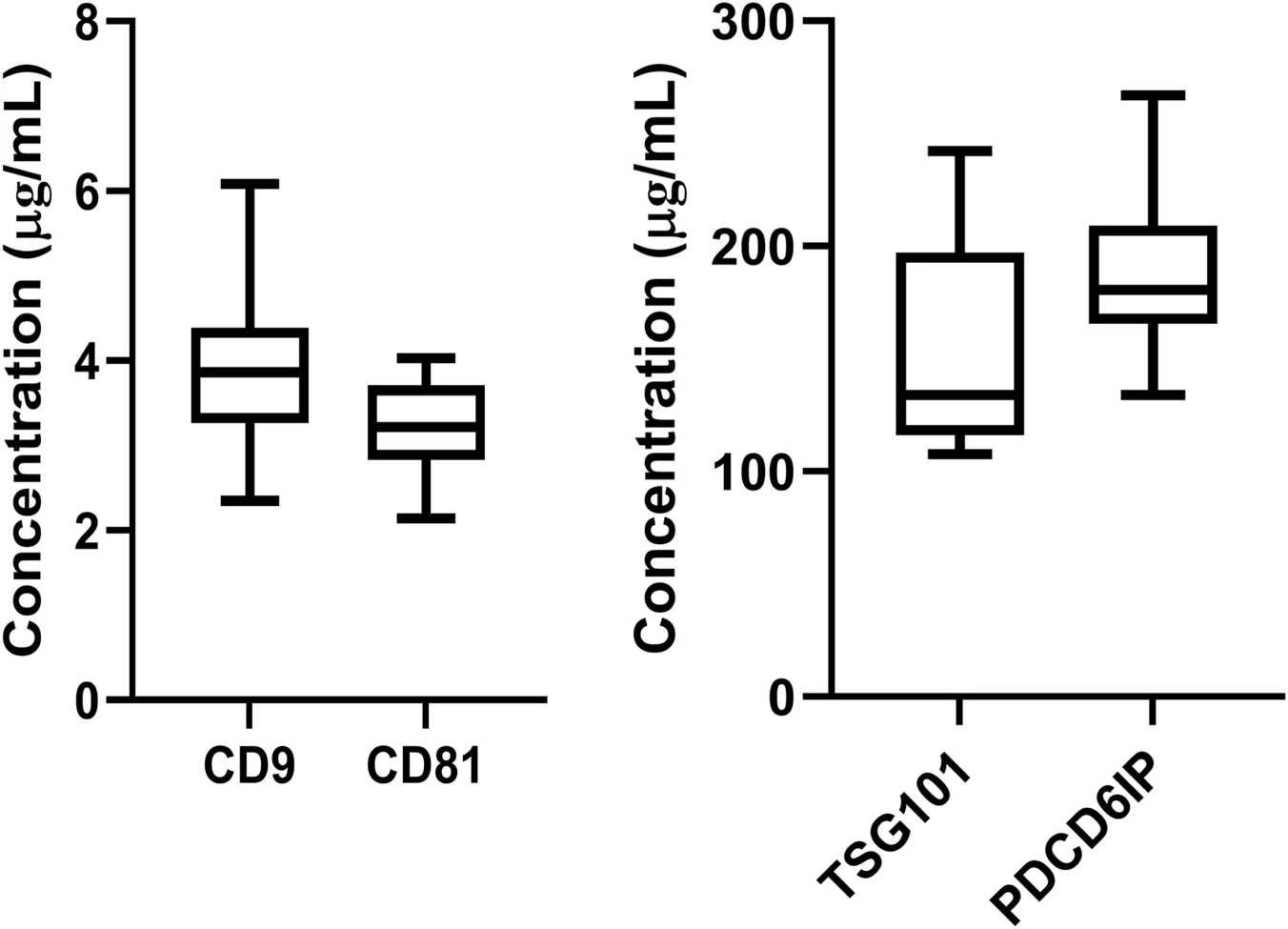
Phenotypic characterization of EVs isolated from OSF samples. Concentration of specific surface markers CD9 and CD81. Concentration of specific cytosol markers TSG101 and PDCD6IP, determined by ELISA. n = 25.

In addition, EVs were analyzed by TEM to assess their morphology (Figure 3). Representative TEM images showed a heterogeneous population of membrane-bound extracellular vesicles with variable sizes. Figure 3 shows a representative immunogold labeling image for CD63. Electron-dense gold particles associated with the vesicle surface indicate the presence of CD63, a well-established EV marker.

**FIGURE 3:**
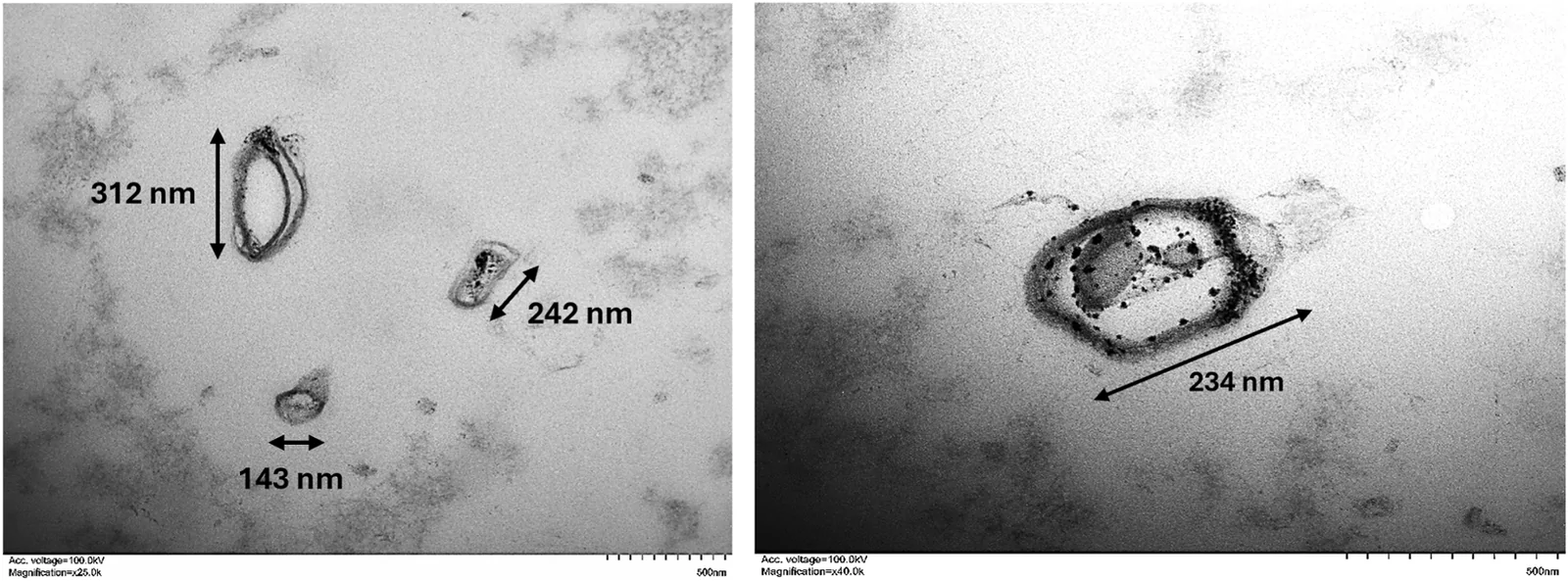
Representative images of EVs isolated from OSF samples by TEM. Cross-section at 25 k magnification of EVs. Longitudinal section with a 40 k magnification of an EV with gold immunostaining of marker CD63. (Acceleration voltage = 100.0 kV).

### 3.3 Optimization of the experimental conditions on OA synoviocytes

Initially, the effects of cell passage and IL-1β stimulation time were evaluated to establish the optimal experimental conditions for primary OA synoviocytes. Figure 4 shows MTT assay results indicating that OA synoviocytes at passage 1 (P1), exhibited significantly higher viability than cells at P0 and P2. Based on these results, subsequent experiments were performed using P1 synoviocytes.

**FIGURE 4:**
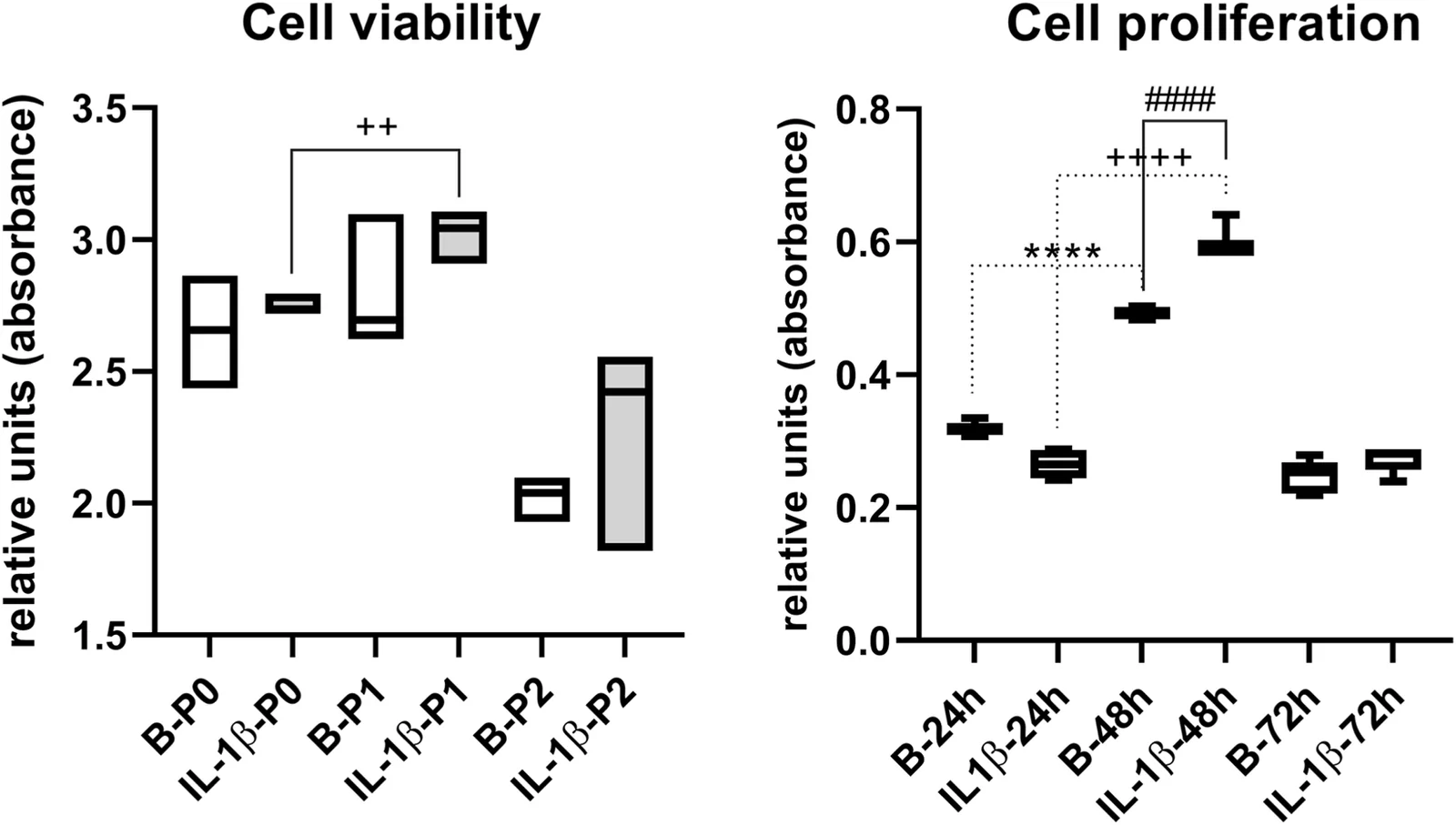
Viability and proliferation of OA synoviocytes in primary culture. Cell viability assessed by MTT assay at different passages (P): P0 (directly isolated synoviocytes), P1, and P2. Effect of IL-1β (10 ng/mL) stimulation time on cell proliferation in P1 synoviocytes assessed by BrdU. ^++^ *p* < 0.01 vs. IL-1β-P0. ^****^ *p* < 0.0001 vs. B-24 h; ^++++^ *p* < 0.0001 vs. IL-1β −24 h; ^####^ *p* < 0.0001 vs. B-48 h. Data are presented as median and IQR. n = 6.

OA synoviocytes at P1 were then stimulated with 10 ng/mL IL-1β for 24, 48, and 72 h and cell proliferation was assessed using the BrdU assay. As shown in Figure 4, IL-1β induced a proliferative response, with significantly higher proliferation at 48 h than at 24 and 72 h. Furthermore, at 48 h, synoviocytes treated with IL-1β showed significantly higher proliferation than untreated synoviocytes. Based on these findings, 48 h was selected as the primary time point for subsequent experiments with EVs. An additional later time point (96 h) was included to assess potential effects over time.

Subsequently, OA synoviocytes viability was evaluated at P1 in the presence of different concentrations of EVs (EV1, 7.20 × 10^8^ EV/mL; EV2, 7.20 × 10^7^ EV/mL; EV3, 1.80 × 10^7^ EV/mL), with or without IL-1β stimulation. MTT assay results at 48 and 96 h showed a slight increase in cell viability in the presence of EVs and/or IL-1β compared with basal OA synoviocytes; however, no significant differences were observed relative to the IL-1β-treated group at either time point (data not shown). A slight increase in cell viability induced by IL-1β has been previously reported by Inoue et al. (2001).

### 3.4 Functional effects of SF-EVs on OA synoviocytes

#### 3.4.1 Cell proliferation

Regarding proliferation, IL-1β stimulation significantly increased synoviocyte proliferation at both 48 h and 96 h compared to untreated cells. In addition, at the three concentrations tested, EVs significantly decreased IL-1β-induced proliferation, suggesting a potential modulatory effect in the inflammatory context (Figure 5). Interestingly, the effects of EVs on synoviocyte viability and proliferation appeared to be maintained at later time points, supporting a sustained biological activity over time.

**FIGURE 5:**
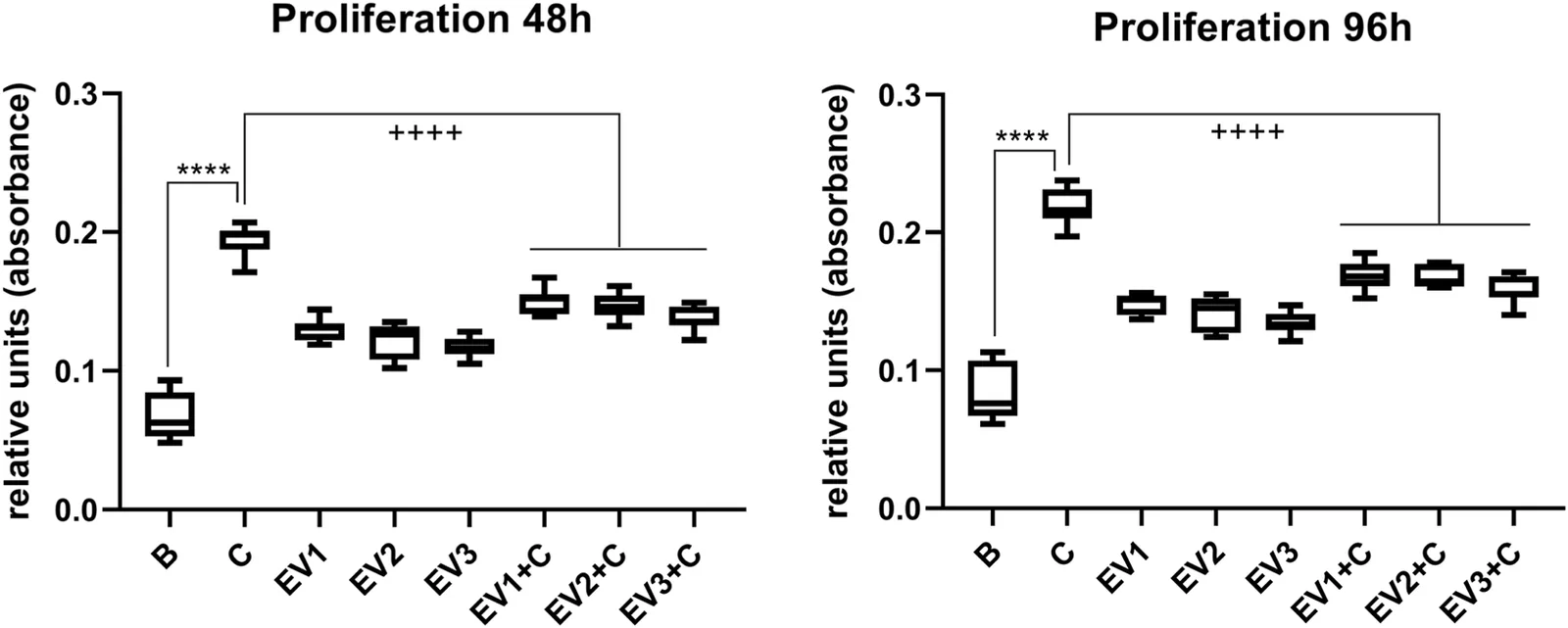
Effects of EVs on proliferation of OA synoviocytes at 48 h and 96 h. B, untreated synoviocytes; C, IL-1β-stimulated synoviocytes; EV1, 7.20 × 10^8^ EV/mL; EV2, 7.20 × 10^7^ EV/mL; EV3, 1.80 × 10^7^ EV/mL ^****^ *p* < 0.0001 vs. B; ^++++^ *p* < 0.0001 vs. C. Data are presented as median and IQR. n = 8.

According to the results obtained, EVs isolated from OSFs samples did not appear to induce cell death, but they reduced the proliferation of IL-1β-stimulated OA synoviocytes. However, considering that these EVs originate from a fundamentally inflammatory environment, we next evaluated whether they also modulated inflammatory and catabolic mediators associated with OA progression.

#### 3.4.2 Effects of SF-EVs on inflammatory, catabolic and homeostatic markers

In this regard, we evaluated the production of one of the most characteristic inflammatory cytokines in OA, IL-6. Figure 6 shows the results after stimulating synoviocytes with IL-1β in the presence or absence of these EVs at 48 and 96 h. As expected, the IL-1β control induced a significant increase in IL-6 compared to baseline. However, EVs significantly decreased this production at all concentrations and time points tested.

**FIGURE 6:**
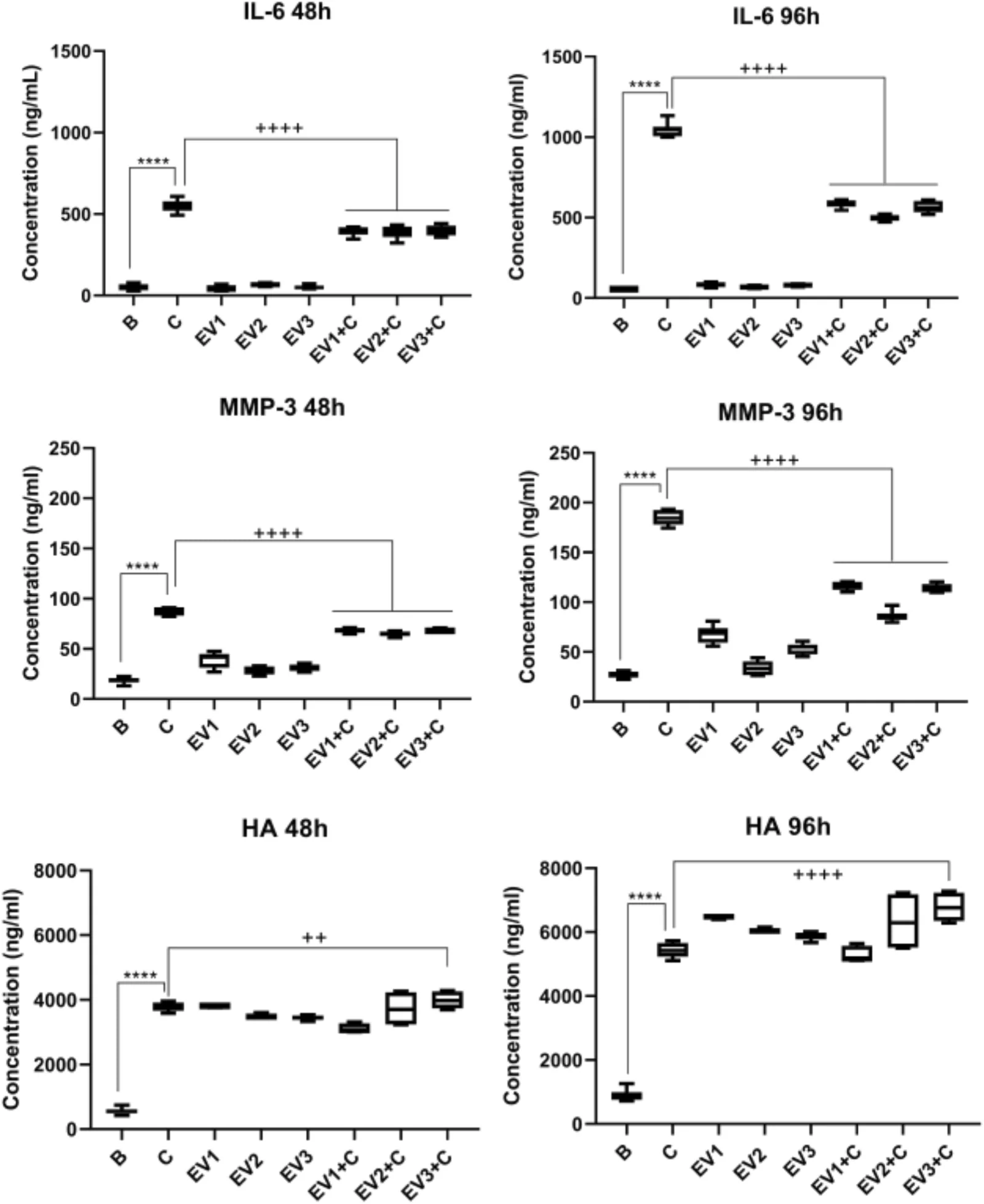
Evaluation of inflammatory markers in treated OA synoviocytes. IL-6, metalloproteinase-3 (MMP-3) and hyaluronic acid (HA) production at 48 h and 96 h by OA synoviocytes. B Untreated synoviocytes; C IL-1β-stimulated synoviocytes; EV1, 7.20 × 10^8^ EV/mL; EV2, 7.20 × 10^7^ EV/mL; EV3, 1.80 × 10^7^ EV/mL ^****^ *p* < 0.0001 vs. B; ^++^ *p* < 0.01 and ^++++^ *p* < 0.0001 vs. C. Data are presented as median (IQR). n = 8.

Regarding matrix metalloproteinase-3 (MMP-3), which is involved in the degradation of the extracellular matrix, we have assessed its production in IL-1β-stimulated OSF cells. Our results indicate that, once again, the three concentrations of EVs derived from OSF samples significantly reduced the MMP-3 levels induced by the IL-1β control. In the absence of IL-1β, the EVs slightly increased MMP-3 production compared to basal conditions in OA synoviocytes (Figure 6).

The hyaluronic acid (HA) content in synovial fluid is considered an important marker of joint integrity. Synoviocytes are cells that produce high levels of this polysaccharide. Therefore, we analyzed HA production by OA synoviocytes under basal and IL-1β-stimulated conditions, both in the presence and absence of EVs derived from OSF samples (Figure 6). The profiles observed at 48 h and 96 h were similar, with higher production of HA at 96 h. Firstly, IL-1β significantly induced HA production compared to unstimulated synoviocytes. Secondly, all EV concentrations tested, both in the absence and presence of IL-1β, also significantly increased HA levels compared to unstimulated cells. Notably, EV3 in the presence of IL-1β significantly increased HA production even compared to IL-1β alone.

As shown in Figure 7, IL-6 and MMP-3 production was higher at 96 h than at 48 h. These results indicate that EVs maintain their ability to inhibit IL-1β-induced effects in OA synoviocytes over time. In contrast, under IL-1β-stimulated conditions, EVs significantly increased HA synthesis by synoviocytes at 96 h compared to 48 h, with a more pronounced effect at the EV3 concentration. These findings suggest a sustained beneficial effect over time.

**FIGURE 7:**
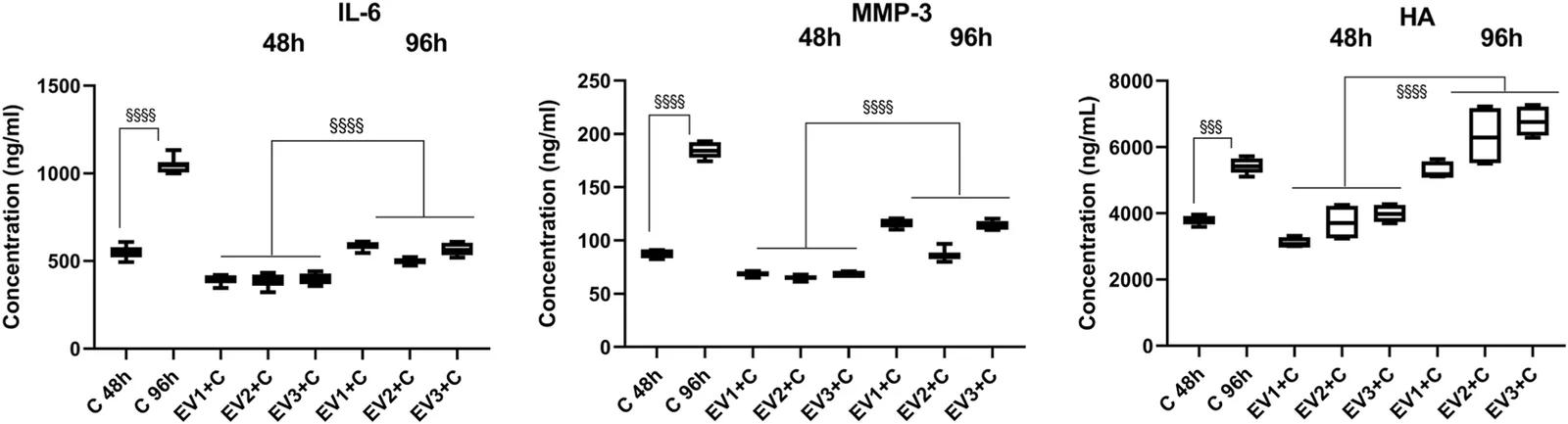
Temporal effects of EVs from OSF on OA synoviocytes at 48 h and 96 h in presence of IL-1β. IL-6, metalloproteinase-3 (MMP-3) and hyaluronic acid (HA) production at 48 h and 96 h. IL-1β-stimulated synoviocytes; EV1, 7.20 × 10^8^ EV/mL; EV2, 7.20 × 10^7^ EV/mL; EV3, 1.80 × 10^7^ EV/mL ^§^^§^^§^ *p* < 0.001 and ^§^^§^^§^^§^ *p* < 0.0001 for comparisons between 48 h and 96 h. Data are presented as median and IQR. n = 8.

### 3.5 Effect of SF-EVs on OA chondrocytes

#### 3.5.1 Cell proliferation

Chondrocytes are the cells responsible for maintaining joint cartilage. However, they also contribute to the production of various soluble factors present in synovial fluid, thereby influencing the overall joint environment. This includes cytokines and matrix metalloproteinases, whereas HA is mainly produced by synoviocytes. Therefore, we also investigated the potential effects of EVs derived from OSF samples on OA chondrocyte cultures. Experiments were performed using P0 chondrocytes, i.e., primary cultures, as these cells exhibit a low physiological proliferation rate (Chu et al., 2025; Liu et al., 2021).

EVs did not significantly modified the proliferation rate of chondrocytes under basal or IL-1β-stimulated conditions (Figure 8). At both 48 h and 96 h, proliferation remained comparable between EV-treated and untreated cells. IL-1β stimulation slightly increased proliferation compared to basal conditions, consistent with its reported proliferative effects. Notably, EV2 concentration significantly reduced proliferation in IL-1β-stimulated chondrocytes compared to IL-1β alone. However, no marked effects on proliferation were observed under the remaining conditions.

**FIGURE 8:**
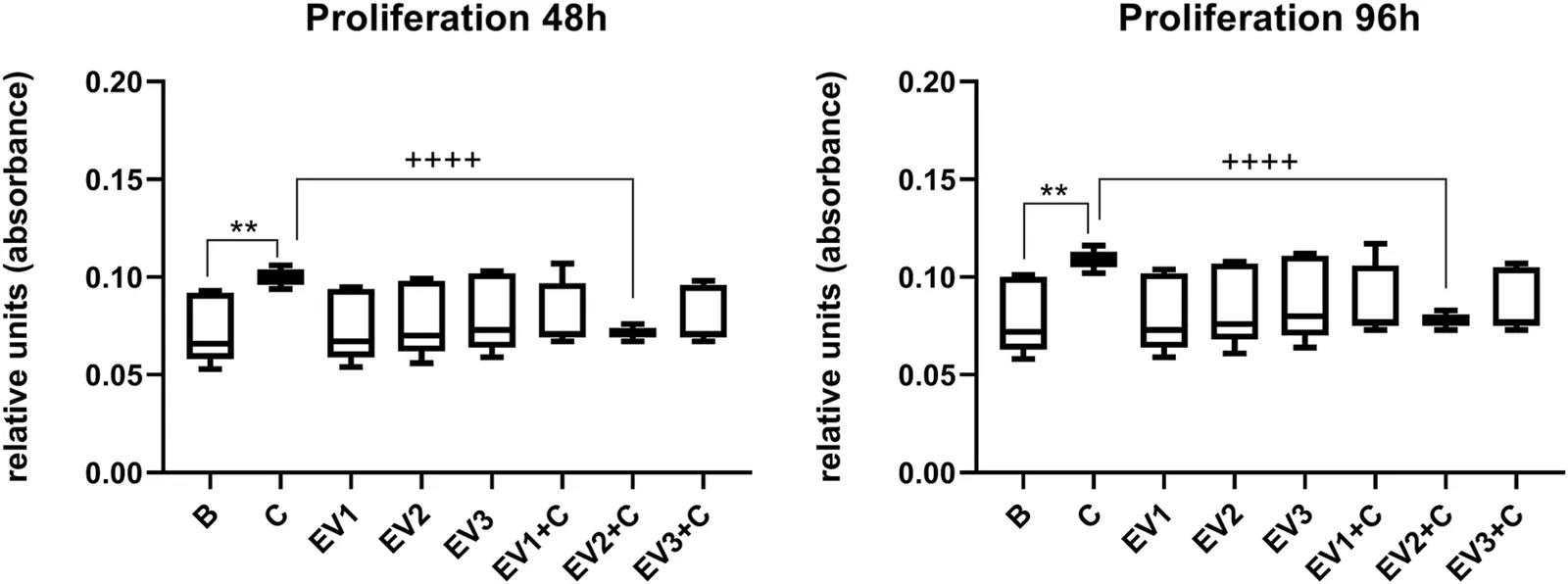
Effects of EVs on the proliferation of OA chondrocytes. Proliferation of OA chondrocytes at 48 h and 96 h. B, untreated chondrocytes; C, IL-1β-stimulated chondrocytes; EV1, 7.20 × 10^8^ EV/mL; EV2, 7.20 × 10^7^ EV/mL; EV3, 1.80 × 10^7^ EV/mL ^**^ *p* < 0.01 vs. B; ^++++^ *p* < 0.0001 vs. C. Data are presented as median and IQR. n = 7.

#### 3.5.2 Effects of SF-EVs on inflammatory, catabolic and homeostatic markers

The results obtained in the analysis of the IL-6 inflammation marker showed that the three concentrations of EV derived from OSF samples significantly reduced IL-1β-induced IL-6 production in OA chondrocytes at 48 h and 96 h (Figure 9). However, IL-1β induced a stronger increase in IL-6 levels at 96 h, resulting in overall higher IL-6 production despite the presence of EVs.

**FIGURE 9:**
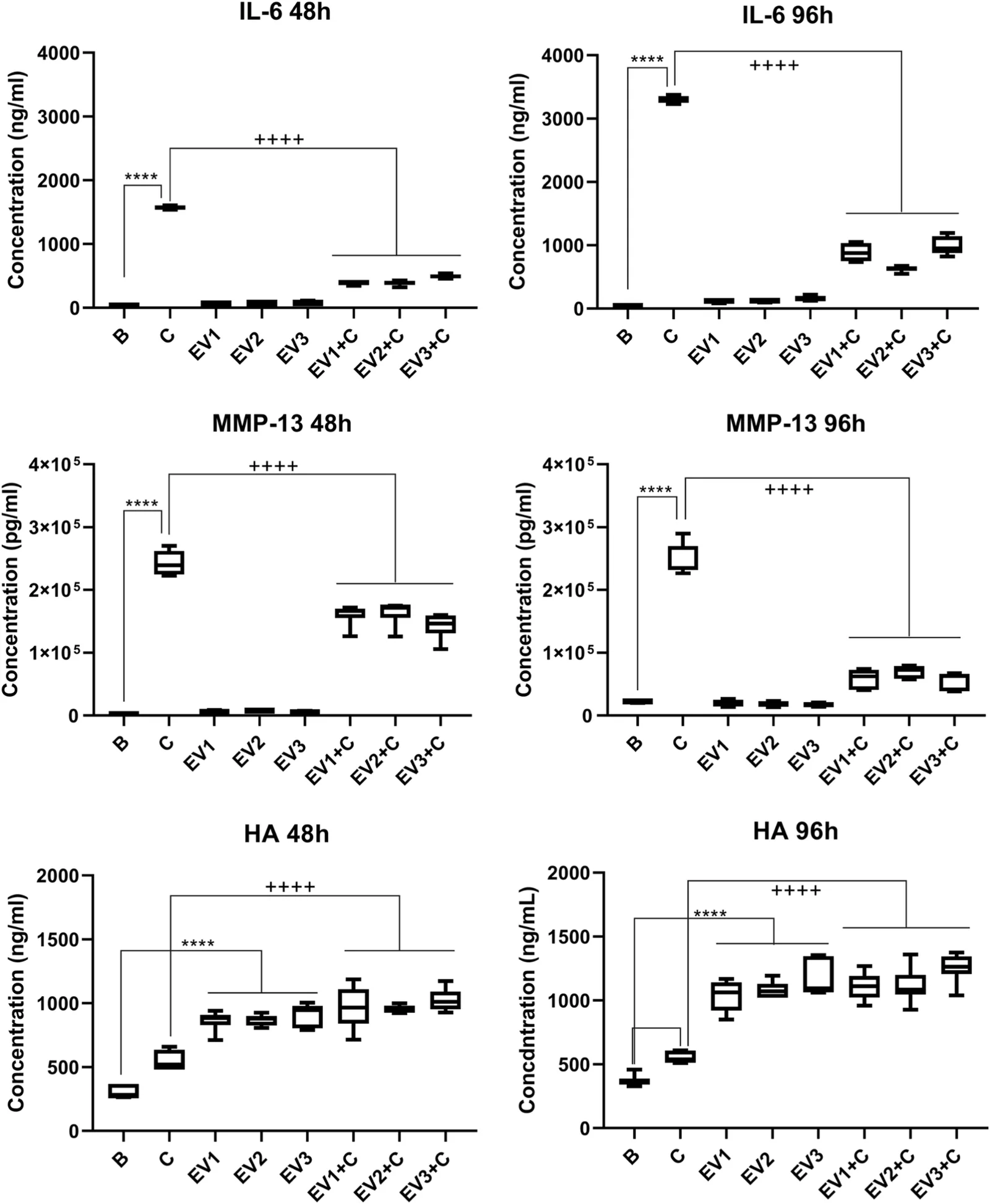
Determination of inflammatory markers in OA chondrocytes. IL-6, matrix metalloproteinase-13 (MMP-13) and hyaluronic acid (HA) production at 48 h and 96 h, respectively. B, untreated chondrocytes; C, IL-1β-stimulated chondrocytes; EV1, 7.20 × 10^8^ EV/mL; EV2, 7.20 × 10^7^ EV/mL; EV3, 1.80 × 10^7^ EV/mL. ^****^ *p* < 0.0001 vs. B; ^++++^ *p* < 0.0001 vs. C. Data are presented as median and IQR. n = 8.

Regarding MMP-13 production, a metalloproteinase responsible for the degradation of type II collagen in cartilage, we observed a significant inhibitory effect of EVs under IL-1β-stimulated conditions at all concentrations and time points. MMP-13 levels induced by IL-1β were similar at 48 h and 96 h. However, the inhibitory effect of EVs appeared more pronounced at 96 h, leading to a greater reduction in MMP-13 levels.

However, our results showed that EVs, both in the presence and absence of IL-1β, significantly increased HA production at all the concentrations and time points tested. These results demonstrate that SF-EVs increase HA production by OA chondrocytes under the experimental conditions evaluated. Whether this increase translates into improved cartilage homeostasis or chondrocyte functionality remains to be established.

To assess whether the effects of SF-EVs were maintained over time, IL-6, MMP-13, and HA levels were compared between 48 and 96 h (Figure 10). IL-6 production in IL-1β-stimulated chondrocytes was significantly higher at 96 h than at 48 h. In the presence of SF-EVs and IL-1β, IL-6 levels were also higher at 96 h than at 48 h.

**FIGURE 10:**
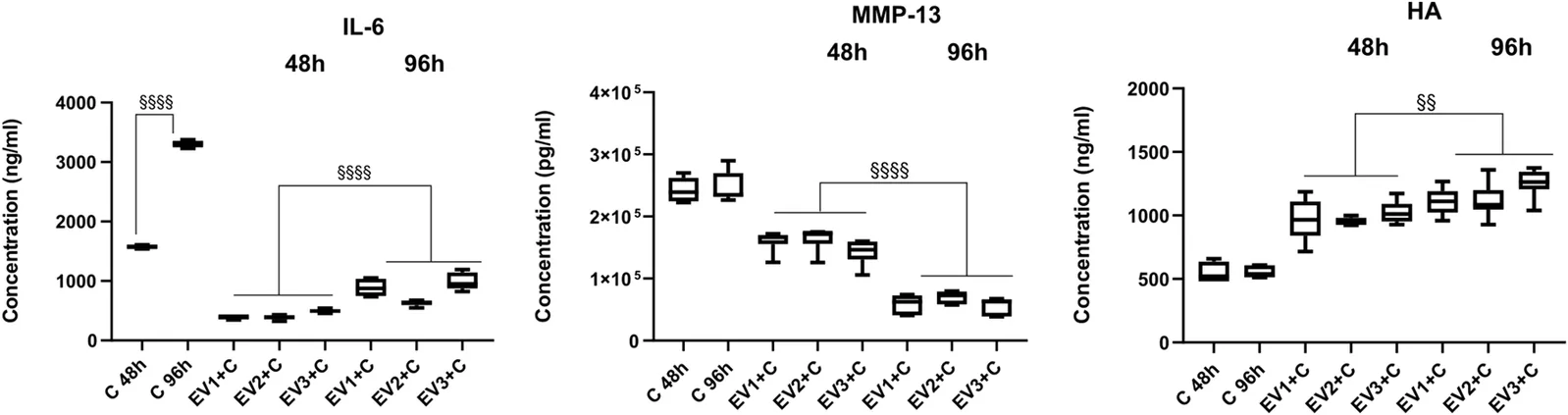
Temporal effects induced by EVs from OSF on OA chondrocytes at 48 h and 96 h in the presence of IL-1β. IL-6, matrix metalloproteinase-13 (MMP-13) and hyaluronic acid (HA) production. ^§^^§^^§^^§^ *p* < 0.0001 for IL-6 and MMP-13 comparisons between 48 h and 96 h; ^§^^§^ *p* < 0.01 for HA comparisons between 48 h and 96 h. Data are presented as median and IQR. n = 8.

In contrast, IL-1β-induced MMP-13 levels were similar at 48 and 96 h. In the presence of SF-EVs and IL-1β, MMP-13 levels were significantly lower at 96 h than at 48 h (Figure 10).

HA levels induced by IL-1β were similar at both time points. However, HA production in the presence of SF-EVs and IL-1β was higher at 96 h than at 48 h, with significant differences observed for the indicated comparisons (Figure 10).

#### 3.5.3 IL-1Ra production

Interleukin-1 receptor antagonist (IL-1Ra) is an endogenous anti-inflammatory cytokine that competitively inhibits IL-1 signaling by binding to the IL-1R1 receptor without activating intracellular pathways, thereby regulating the inflammatory response. We evaluated IL-1Ra production by OA chondrocytes stimulated with IL-1β in the presence and absence of EVs present in OSF. The results indicate that the cytokine IL-1β in the presence of EVs increased IL-1Ra production compared to IL-1β control. These findings demonstrate that SF-EVs increase IL-1Ra production by OA chondrocytes, suggesting that this response may contribute to the modulation of the inflammatory response (Figure 11).

**FIGURE 11:**
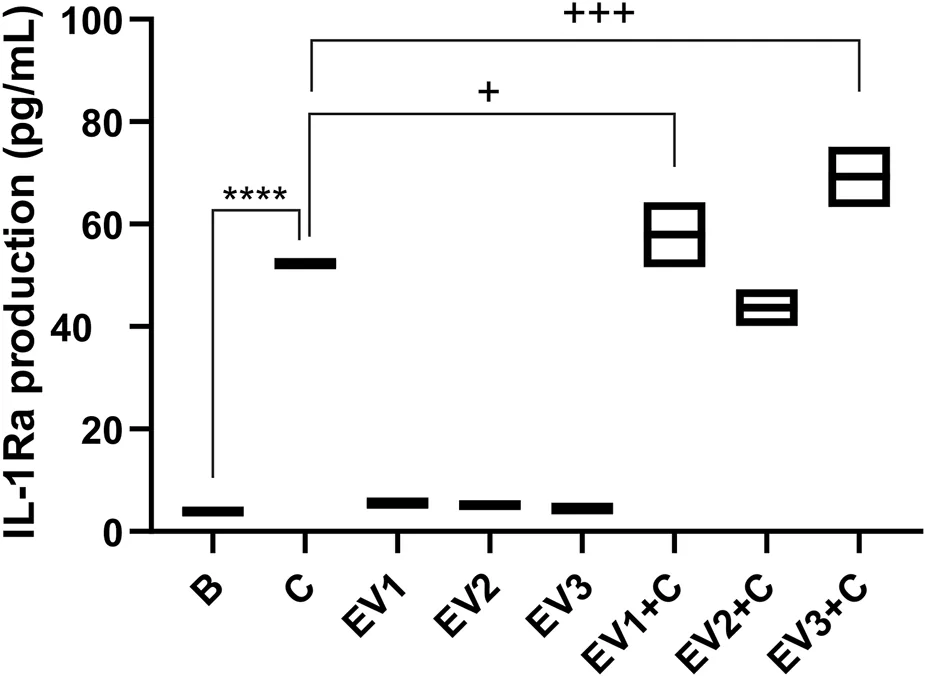
Production of the soluble anti-inflammatory cytokine IL-1Ra by OA chondrocytes. IL-1Ra production at 48 h by OA chondrocytes. B, untreated chondrocytes; C, IL-1β-stimulated chondrocytes; EV1, 7.20 × 10^8^ EV/mL; EV2, 7.20 × 10^7^ EV/mL; EV3, 1.80 × 10^7^ EV/mL ^****^ *p* < 0.0001 vs. B; ^+^ *p* < 0.05 and ^+++^ *p* < 0.001 vs. C. Data are presented as median and IQR. n = 5.

## 4 Discussion

In this study, we investigated the modulatory effects of EVs isolated from the SF of patients with advanced OA on OA synoviocytes and chondrocytes. Experiments were performed using different concentrations of EVs and varying incubation times, aiming to reproduce the inflammatory microenvironment characteristic of OA as closely as possible.

Although numerous studies have explored the role of EVs and microvesicles in joint diseases (particularly rheumatoid arthritis) (Liu et al., 2026; Jin et al., 2025) there is still limited evidence specifically focused on OA (Yang et al., 2023; Sun et al., 2025). In some cases, findings from rheumatoid arthritis have been extrapolated to OA, despite their distinct aetiologies and progression mechanisms (Zhang et al., 2024; Oveisee et al., 2024; Caliogna et al., 2024). In the present study, only OA patient samples were included. Biochemical and macroscopic characterization of SF samples excluded arthritic conditions. The samples displayed features consistent with OA, including a transparent to slightly turbid appearance, medium-to-high viscosity, and very low or undetectable levels of C-reactive protein. Inflammatory profiling revealed elevated levels of IL-1β and IL-6, confirming the inflammatory status of OSF samples (Mihailova, 2022). At the same time, hyaluronic acid (HA) concentrations were also high, consistent with the viscosity observed in most samples.

The SF-derived EV-enriched preparations were characterized according to current extracellular vesicle guidelines, including particle size determination, assessment of EV-associated surface and cytosolic markers, and morphological evaluation by TEM (Théry et al., 2018; Welsh et al., 2024; Coumans et al., 2017). However, this characterization does not allow definitive assignment of an exclusively endosomal origin or the identification of the cellular sources contributing to the isolated EV population.

Functionally, we demonstrated that EVs significantly inhibit the proliferation of IL-1β-stimulated OA synoviocytes. This finding is particularly relevant, as synovial hyperplasia and pannus formation are key contributors to OA progression (Cinar, 2025; Semenistaja et al., 2025; Furuzawa-Carballeda et al., 2008). Increased synoviocyte proliferation enhances joint inflammation and tissue damage (Cinar, 2025; Semenistaja et al., 2025); therefore, targeting this process may represent an effective strategy to limit disease progression. In addition, synoviocytes are major producers of inflammatory mediators such as IL-6 and matrix-degrading enzymes including MMP-3 (Machado et al., 2019). Our results show that EVs reduce IL-1β-induced production of both IL-6 and MMP-3, with a stronger effect at early time points that is maintained over time. These findings suggest that SF-derived EV-enriched preparations may have anti-inflammatory properties under the experimental conditions evaluated. However, further *in vivo* studies are required to determine their therapeutic relevance. We also observed that EVs increased HA production in synoviocytes compared to IL-1β stimulation alone, with more pronounced effects at longer incubation times. Maintenance of HA levels in synovial fluid is critical for preserving joint homeostasis and limiting cartilage degradation (Le-Chan et al., 2026).

Chondrocytes, which exhibit low proliferative capacity (Chu et al., 2025; Liu et al., 2021), did not show significant changes in proliferation upon IL-1β stimulation, either in the presence or absence of EVs. Notably, only the EV2 concentration tested in combination with IL-1β led to a significant reduction in proliferation compared to IL-1β alone. Analysis of inflammatory and catabolic markers revealed that both IL-6 and MMP-13 production were reduced in chondrocytes treated with EVs at 48 and 96 h compared to IL-1β controls. In parallel, EVs significantly increased HA synthesis in chondrocytes, both under basal and inflammatory conditions. Longer incubation times were associated with stronger inhibition of MMP-13 and a progressive increase in HA production. Together, these results suggest sustained anti-inflammatory and anticatabolic effects of SF-derived EV-enriched preparations in OA chondrocytes under the experimental conditions evaluated.

The ability of SF-derived EV-enriched preparations to enhance HA production in both synoviocytes and chondrocytes represents an interesting biological finding. However, only total HA production was evaluated in the present study. Since the biological properties of HA are strongly influenced by its molecular weight and structural integrity, the functional significance of the observed increase cannot be established from the present data. Therefore, further studies are required to determine whether this response contributes to joint homeostasis or has potential biological or clinical relevance.

It should be noted that the EVs isolated from OSF originate from an inflammatory microenvironment. This environment contains both pro-inflammatory and degradative mediators, as well as anti-inflammatory and regenerative factors acting as compensatory mechanisms. The SF-derived EV-enriched preparations analysed in this study likely represent a heterogeneous mixture of EVs released by multiple joint-resident cell populations, including synoviocytes, chondrocytes, immune cells, vascular cells and potentially circulating cells. Consequently, the biological responses observed in the present study should be interpreted as the net functional effect of this heterogeneous EV preparation rather than the activity of a specific EV subtype. This heterogeneity complicates the interpretation of the molecular mechanisms underlying the observed biological effects. For instance, IL-1 induces the production of IL-1 receptor antagonist (IL-1Ra) as a negative feedback mechanism that limits inflammation by blocking IL-1β receptor signaling (Mohd Noor et al., 2025; Palmer et al., 2002; Murata et al., 2003). In this study, we evaluated the capacity of OA chondrocytes to produce IL-1Ra in response to IL-1β in the presence or absence of EVs. Our results show that EVs significantly increase IL-1Ra production under inflammatory conditions, particularly at the EV1 and EV3 concentrations. This finding raises the possibility that increased IL-1Ra production may contribute to the anti-inflammatory profile observed. However, the molecular mechanisms responsible for this effect were not investigated in the present study.

However, the literature reports conflicting findings regarding the role of EVs derived from inflammatory environments. Some studies suggest that these EVs enhance catabolic and inflammatory responses. For example, EVs derived from IL-1β-stimulated fibroblast-like synoviocytes have been shown to exacerbate inflammatory responses in healthy chondrocytes (Kato et al., 2014). However, another study conducted in chondrocytes has shown that, compared to cells cultured under normal conditions, chondrocytes exposed to an inflammatory environment can alter the protein cargo of their EVs (Liu et al., 2020). In this context, EVs derived from primary chondrocytes have been reported to interact with damaged mitochondria, restoring their structure and function, thereby reducing reactive oxygen species production and increasing intracellular adenosine triphosphate (ATP) levels, ultimately improving chondrocyte energy metabolism. Furthermore, these EVs promote the polarization of macrophages toward an anti-inflammatory M2 phenotype while reducing the proportion of pro-inflammatory M1 macrophages, thereby modulating the immune response within the joint and slowing the progression of OA (Zheng et al., 2019).

Additional evidence indicates that EVs from fibroblast-like synoviocytes may increase senescence and inflammation in these cells while exerting beneficial effects on macrophage-like synoviocytes. Wang et al. reported that EVs derived from fibroblast-like synoviocytes express miR-146 via activation of the Toll-like receptor 4 (TLR4)/TNF receptor-associated factor 6 (TRAF6)/nuclear factor kappa B (NF-κB) signaling pathway (Wang et al., 2024). These EVs promote the polarization of synovial macrophages from the pro-inflammatory M1 phenotype toward the anti-inflammatory M2 phenotype, thereby reducing cartilage damage and synovial hyperplasia.

Consistent with these discrepancies, our previous work demonstrated that EVs derived from IL-1β-stimulated human monocytes induce pro-inflammatory and catabolic effects in primary OA chondrocytes (Carrabs et al., 2024). Similarly, Domenis et al. reported that EVs isolated from OSF induce the production of multiple inflammatory factors in M1 macrophages (Domenis et al., 2017). Importantly, these findings highlight that EV-mediated effects are highly dependent on the target cell type. As the aforementioned study focused specifically on macrophage responses, the observed effects may be influenced by cell-specific factors. Indeed, different cell types may respond differently to similar stimuli, underscoring the importance of cellular context when interpreting these results.

In agreement with this, we found differential responses between synoviocytes, and chondrocytes exposed to the same pool of EVs. Moreover, both cell types are themselves active sources of EVs, further contributing to the complexity of intercellular communication within the joint microenvironment.

The present study demonstrates that heterogeneous SF-derived EV-enriched preparations isolated from patients with OA modulate the inflammatory response of primary synoviocytes and chondrocytes under IL-1β-stimulated conditions. These findings reflect the overall biological activity of the heterogeneous EV preparations analysed and should not be interpreted as evidence for the effects of a specific EV subtype or cellular source. SF-EVs reduced the production of inflammatory and catabolic mediators while increasing hyaluronic acid synthesis in both cell types and enhancing IL-1Ra production in chondrocytes, supporting an overall anti-inflammatory and anticatabolic profile within the osteoarthritic joint microenvironment.

The *in vitro* IL-1β-stimulated model used in this study does not fully reproduce the complexity of the osteoarthritic joint environment. Associations between radiographic disease severity, synovial fluid inflammatory profile, and EV concentration, molecular composition, or biological activity were not evaluated. Moreover, EV cargo and mechanistic pathways were not characterized, precluding identification of the molecular mediators responsible for the observed effects.

EV characterization and isolation also present certain methodological limitations. EV-associated markers were assessed by ELISA in the whole preparation rather than by single-particle or particle-based approaches, and process controls such as PBS-loaded SEC columns, blank fractions, or EV-depleted synovial fluid fractions were not included. In addition, quantitative assessment of non-vesicular components, including total protein concentration, particle-to-protein ratio, albumin, or ApoA1, was not performed. Therefore, a contribution of residual co-isolated non-vesicular components to the observed biological effects cannot be completely excluded.

The 0.22-µm filtration step was intended to remove large particles rather than to provide an absolute size cut-off; thus, particles with apparent diameters above 220 nm detected by TRPS may reflect EV deformability, filter pore-size variability, particle aggregation, or methodological limitations of TRPS. Finally, two commercially available SEC platforms were used for EV isolation without a formal comparison of the resulting preparations, and potential differences related to the isolation workflow therefore cannot be excluded.

Despite these limitations, our findings provide new evidence supporting the functional role of endogenous SF-derived EVs in OA. Future studies should investigate their molecular cargo and mechanisms of action, evaluate their relevance *in vivo*, and determine whether specific EV populations contribute differentially to joint inflammation and tissue homeostasis.

## Data Availability

The original contributions presented in the study are included in the article/Supplementary Material, further inquiries can be directed to the corresponding authors.
